# Validated method for polystyrene nanoplastic separation in aqueous matrices by asymmetric-flow field flow fraction coupled to MALS and UV–Vis detectors

**DOI:** 10.1007/s00604-023-05851-7

**Published:** 2023-07-07

**Authors:** Iris H.Valido, Victor Fuentes-Cebrian, Alba Hernández, Manuel Valiente, Montserrat López-Mesas

**Affiliations:** 1 GTS Research Group, Department of Chemistry, Faculty of Science, Universitat Autònoma de Barcelona, Cerdanyola del Vallès, 08193 Barcelona, Spain; 2 Group of Mutagenesis, Department of Genetics and Microbiology, Faculty of Biosciences, Universitat Autònoma de Barcelona, Cerdanyola del Vallès, 08193 Barcelona, Spain

**Keywords:** Nanoplastics, AF4-MALS-UV, Validation, Polystyrene, Spheres, Reference standards

## Abstract

Plastics with nanosize (nanoplastics, NPLs) must be characterized, since they can be toxic or act as carriers of organic and inorganic pollutants, but there is a lack of reference materials and validated methods in the nanosize range. Therefore, this study has focused on the development and validation of a separation and size characterization methodology of polystyrene latex nanospheres, by using an asymmetric-flow field flow fraction system coupled to multi-angle light scattering and ultraviolet–visible detectors (AF4-MALS-UV). Hence, this work presents a fully validated methodology in the particle size range 30 to 490 nm, with bias between 95 and 109%, precision between 1 and 18%, LOD and LOQ below 0.2 and 0.3 µg respectively, except for 30-nm standard, for both detectors, and showing stable results for 100 analyses.

**Graphical Abstract:**

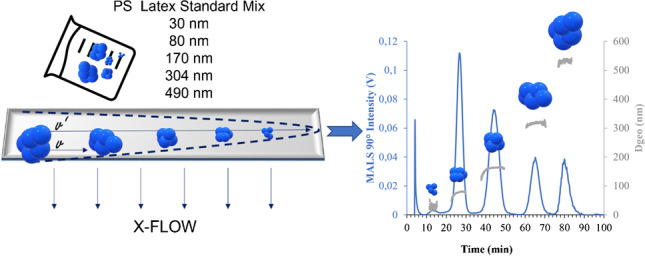

## Introduction

Plastics are multi-functional materials used for a variety of purposes, from food preservation to medicine supplies or clothing. Due to this large usage nowadays, its global production has increased from 250 million metric tons (Mt) in 2009 to 367 million Mt in 2020, being China the major producer (32%) and with Europe producing around 15% [1]. Considering the rising use tendency and the lack of proper residue management, it has been estimated that 33,000 MT of plastic waste being generated by 2050, from which only the 27% will be recycled, the 36% will be incinerated, and the final 37% will be disposed into the environment [2].

The large and growing use, together with the waste mismanagement, of the plastic products has impacted the environment in many ways, as being disease vectors, marine, soil, and air pollutants, even impacting global warming, among others [3]. Plastic without any type of major degradation can cause physical damage to marine creatures mainly, due to its form by getting stuck between extremities, hindering the movement, or in the mouth, making impossible animals to eat [4]. Furthermore, when this waste suffers from physical and chemical weathering, their size reduces, forming particles from 1 µm to 5 mm, called microplastics (MPLs) [5], which can enter the animal’s system, making this material a much more dangerous pollutant. Microplastics can act in two different ways in the animal’s body, the first one is to induce physical injuries in the digestive tract that can lead to major problems from digestive or endocrinal harm to death [6]. Moreover, they can also act as carriers of organic such as polychlorinated biphenyls or bisphenol A [7], which have been reported to have toxic and mutagenic activities in several marine creatures and being some of the most common organic pollutants in the marine ecosystem, as well as metals [8, 9]. Nevertheless, plastics and microplastics can also endure more weathering until they reach smaller sizes being equal or less than 1000 nm, forming what it is called nanoplastics (NPLs) [10]. Human exposure to microplastics and nanoplastics (MNPLs) occurs directly through ingestion, inhalation, and dermal contact but there is a lack of human biomonitoring studies [11]. Nanoplastics and their potential hazard come mainly from their size, since they are in the nanoscale range, they are able to trespass the blood–brain barrier [12, 13], which presents a new risk for living beings as this pollutant can easily enter to the bloodstream and cellular medium [14]. In the latest years, several investigations have reported the toxicity of the NPLs themselves in a variety of animals, mostly, aquatic specimens [15]. Like microplastics, nanoplastics can act as potential carriers of several types of pollutants, such as plastic additives, heavy metals [9, 16], or volatile organic compounds [17], and due to their size, they will be able to enter the cells without any physical harm and, potentially, release those compounds into the intracellular medium [18].

**Fig. 1:**
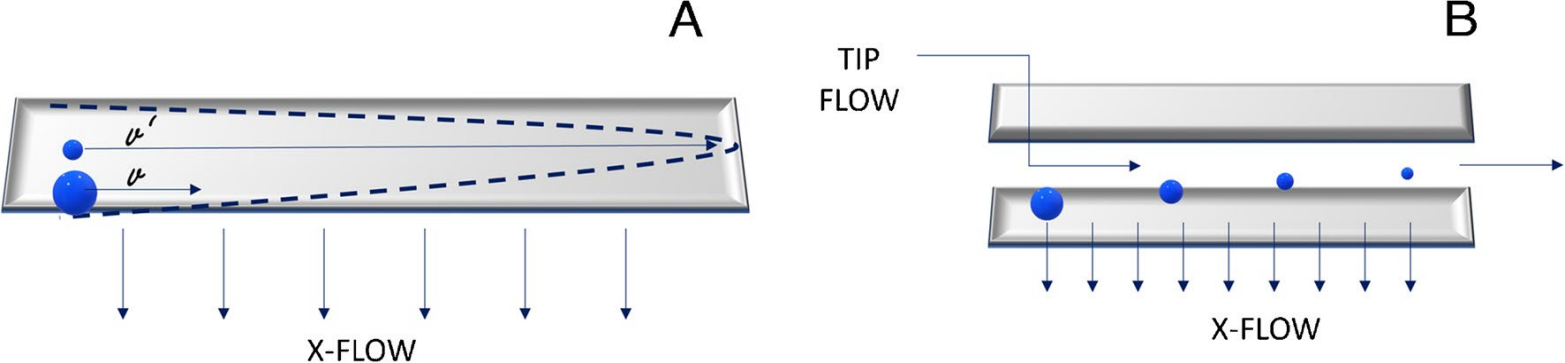
**A** Illustration of the parabolic flow and the diffusion difference among particle size. **B** Illustration of the channel and the two main flows involved in the creation of the parabolic flow

This type of materials can be detected in several ways depending on its size, being microplastics mostly analyzed by the naked eye, dividing by color and size or by microscopy if the size is smaller [19, 20]. Nevertheless, due to the different behaviors of NPLs, when compared to MPLs, methodologies already described in the literature for MPLs cannot be directly applied. Furthermore, a common technique used in the characterization of these pollutants’ composition is pyrolysis gas chromatography mass spectrometry (Pyr-GC–MS). This technique fragments the plastic by applying high temperature and analyzes the volatile molecules generated, which are characteristic compounds of the plastic or other pollutants adsorbed on it [21, 22]. On the other hand, non-destructive techniques are also used in this field, as attenuated total reflectance fourier infrared spectroscopy (ATR FT-IR) which detects the characteristic vibrations of specific regions of the analyte molecule [23]. On the other hand, due to the increasing interest in the characterization of NPL size, it has become a new path of research in many areas. Recently, the European Commission has published a document showing the state of knowledge about NPLs and the challenges to be faced in order to stablish a new policy regarding NPLs [24]. This type of material is usually characterized using tools such as scanning electron microscopy, which can approximately determine the size of a nanoscale material by irradiating the sample with a high energy beam and scanning the backscattering electrons to integrate this signal into an image [25]. Moreover, dynamic light scattering (DLS) or multi-angle dynamic light scattering (MALS) is also used when determining the size of NPLs by detecting the scattered light by the sample into a certain angle, or multiple angles, and transforming its intensity to a size [26]. Both techniques work with static samples, which makes it difficult to obtain a full-size characterization of the analyte, mainly due to shielding effect of larger molecules or particles to smaller ones [27]. However, this problem can be solved by the application of asymmetric flow field flow fractionation (AF4), which allows to separate nanoparticles by size inside a channel. This technique bases its separation on the creation of a parabolic flow (Fig. 1A) by the combination of a parallel flow (TIP Flow) and a vertical flow (X-Flow) to the separation channel (Fig. 1B), which originates different velocities along the height of the channel, being the fastest one in the middle [28]. Considering that smaller particles have a higher diffusion coefficient, these will diffuse to the center of the channel acquiring larger velocities and eluting faster, and on the contrary, bigger particles will have lower velocities due to its low diffusion, which will result in larger elution times [29].

The AF4 technique is usually coupled to two types of detectors, concentration detectors, and size detectors. An example of concentration detector is the ultraviolet–visible spectrophotometers (UV). On the other hand, size detectors such as MALS can directly detect the size of a sample by its irradiation with a beam and the detection of the scattered light into the different detectors situated at known angles [30]. Gyration radius (*R*_g_) can be obtained using a modified static light scattering equation, to be later transformed into geometric diameter (*D*_geo_) by the fitting and approximation depending on the geometry of the particle [31], as shown in Eq. 1, being 0.775 the typical form factor of a sphere.

1 $${D}_{\mathrm{geo}}=\frac{{R}_{\mathrm{geo}}}{0.775}\cdot 2$$

Although there are publications that explore the use of the AF4 to obtain size information of NPLs [32, 33], there is still a lack of validated methodologies to apply in this field [11, 24], also for the validation of standards in aqueous solution. Reference materials are being developed and validated methodologies; alternatives to SEM and TEM are needed for their measurement. The main objective of this research is to develop and validate an AF4-MALS-UV method for the determination of a wide range of nanoplastic sizes of standards in aqueous solutions.

## Materials and methods

### Reagents and standard preparation

Polystyrene (latex) spheres SEM/TEM standards (Std.) (Ted Pella, Inc., Redding, CA), provided in an aqueous stabilized solution, for non-aggregation purpose, at a concentration of 0.1% (w/v) were used to prepare a standard mix containing a combination of diameters: 40 ppm of 30 ± 9 nm and 80 ± 14 nm, 8 ppm of 170 ± 9 nm and 304 ± 9 nm, and 6 ppm of 490 ± 15 nm. To avoid the aggregation of the NPLs, the Std. mix was prepared using NovaChem Surfactant 100 (IESMAT, Madrid, Spain) 0.2% (v/v).

Polystyrene (latex) Spheres NIST standards (IESMAT, Madrid, Spain) of certified mean diameter (and nominal diameter): 62 ± 3 nm (60 nm), 122 ± 3 nm (125 nm), and 345 ± 7 nm (350 nm), at a concentration of 1% (v/v) in an aqueous stabilized solution were used for the bias test.

All solutions were prepared using “ultrapure type 1” water (Milli-Q, Millipore, 18.2 mS cm^−1^).

### Equipment

The NPL separation was performed using an AF4-MALS-UV. The equipment consists of an AF2000 Asymmetric Flow FFF (AF4) system with two PN1130 Isocratic Pumps, a Kloehn V6 Pump, a PN5300 Autosampler, a PEEK AF4 Channel with a 10 kDa regenerated Cellulose membrane, and a 350 µm height spacer. The mobile phase used for the separation methodology was the same used for the standard preparation, NovaChem Surfactant 100 at 0.2% (v/v). The spectral data was provided by a PN3212 UV–VIS detector at 248 nm. Moreover, a PN3609 MALS detector with 9 angles provided the *R*_g_ by applying the sphere fitting provided by the AF2000 Software. All the equipment mentioned above was provided by Postnova Analytics GmbH, Germany. The *D*_geo_ of the NPLs was obtained applying Eq. 1.

### Method validation

The method validation performance and ranges of acceptance from EURACHEM guidelines [34], Food and Drug Administration [35], and ISO/TS 21362:2018 [36] were followed. The validation parameters optimized studied were accuracy (as precision and bias), linearity, limit of detection (LOD), limit of quantification (LOQ), and resolution.

#### Precision

Repeatability was studied by injecting five times a volume of 25 µL of the standard mix. The retention times (*t*_r_) for both, UV and MALS, detectors were compared between them, and the results were expressed in RSD (%).

Intermediate precision was studied by injecting five times a volume of 25 µL of the standard mix, two different days by the same analyst. The variance and means between days of the retention times and *D*_geo_ of each detector, UV and MALS, were studied by *F*-test and *t*-test: two-sample assuming equal variances, and the results were expressed in RSD (%).

#### Bias

The bias was evaluated to assess trueness [32]. It was tested injecting five times a volume of 25 µL of the NIST standard mix. The average geometric diameter (Av. Experimental *D*_geo_) calculated from MALS data were compared to the theoretical ones (*D*_geo_) and the results were expressed as apparent recovery (*R*, %), following Eq. 2.

2 $$R\;\left(\%\right)=\frac{\mathrm{Av}.\mathrm{ Experimental\;}{D}_{\mathrm{geo}}}{{D}_{\mathrm{geo}}}\cdot 100$$

#### Linearity, LOD, and LOQ

These parameters were studied by the injection of several volumes of the standard mix from 5 to 250 µL. The results were expressed in injected mass (µg) vs. intensity 90º MALS (V), injected mass (µg) vs. intensity UV (V), and injected mass (µg) vs. Area UV. Linearity of the results was considered where the overall studied points expressed a linear fitting with an *R*^2^ parameter higher to 0.99 [35]. Furthermore, the limit of detection (LOD) was considered as the point of least injected mass where NPLs could be detected, considering three times the signal to noise ratio of the blank (*σ*_Blank_) of each of the detectors and substituting each of the results in the corresponding calibration curve to obtain the minimum injected mass able to be detected.

The limit of quantification (LOQ) was considered as the point of least injected mass where the intensity was above ten times the signal to noise ratio.

#### Resolution

This parameter was calculated in both detectors at the same time taking into account the retention times of consecutive peaks using the peak full width at half maximum.

#### Additional studied parameters

Two additional parameters were studied: membrane saturation and injection volume. The membrane saturation was tested by comparing five injections of 25 µL of standard mix in a 100-h-of-use cellulose membrane with five injections of 25 µL of standard mix in a new cellulose membrane. To do so, *F*-test and *t*-Student test were performed, and bias and precision were compared. On the other hand, the mass injected precision was tested by comparing five injections of 25 µL of standard mix with five injections of 50 µL of the same standard mix. To do so, *F*-test and *t*-Student test were performed, and accuracy of the retention times of the UV detector and the *D*_geo_ of the MALS detector were compared.

## Results and discussion

### Optimized AF4 analysis method

After the optimization of the different parameters, the final developed method used for the separation of polystyrene nanospheres NPLs by AF4-MALS-UV is presented in Table 1.

**Table 1:** Optimized parameters for the separation of polystyrene nanospheres by AF4-MALS-UV

	Tip flow (mL/min)	Focus flow (mL/min)	Cross-flow (mL/min)	Time (min)	Function type
Focusing step	1.5	0.2	1.2	3	-
Separation step	2.0	0	1.5	0.2	Constant
	2.0 to 1.5	0	1.5 to 1.0	10	Linear
	1.5 to 0.5	0	1.0 to 0	80	Exponential (0.7)
Cleaning step	0.5	0	0	20	Constant

**Fig. 2:**
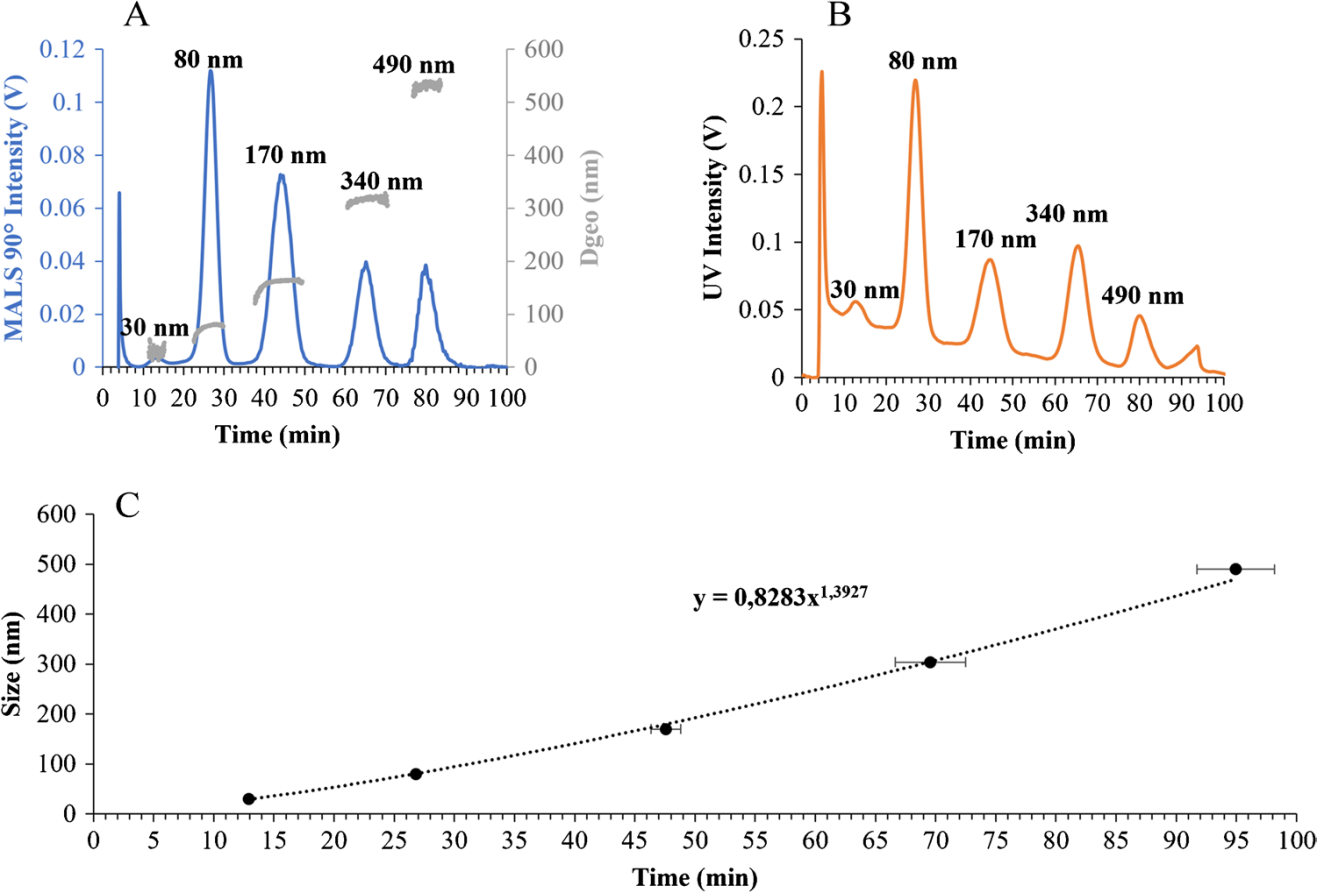
AF4 fractograms of the Standard mix separation with their respective retention times (from lower to higher NPL size) measured by **A** MALS detector at 90° and **B** UV detector; and **C** exponential regression of the retention time calibration

An example of the results obtained with the optimized method for the analysis of NPLs Std. mix in aqueous matrix can be seen in Fig. 2. The MALS detector presented a linear signal with no affectation by the pressure changes in the pumps (Fig. 2A). However, the UV detector showed noticeable changes in its baseline signal due to the pressure changes in the system (Fig. 2B). The retention times obtained from both detectors showed a clear exponential fitting (*r*^2^ > 0.99) when plotted against standard size (Fig. 2C), which results from the exponential of the separation step programmed in the method described in Table 1.

### Method validation

#### Precision

Repeatability was studied for the *D*_geo_ and *t*_r_ for MALS and UV detectors, respectively. The results (Table 2, top) confirmed that both detectors showed a repeatability within range of acceptance (RSD = 1–20%) [33, 35]. A noticeable tendency is the increasing precision in the MALS detector, with the increase of the standard size, agreeing with the increasing sensibility of the equipment for higher sizes. On the contrary, UV detector precision tends to decrease as the retention time increases, this can be due to the lower mobility of higher size standards which increases the dispersion of the peaks, leading to a lower precision. These results imply that there are no statistical differences between the obtained using the optimized method for analyzing NPLs within a day.

**Table 2:** Repeatability (top) and intermediate precision (bottom) results from MALS and UV detectors using the NPL standard mix

	Std. D_geo_	MALS detector			UV detector		
		Average D_geo_ (nm)	Std. Dev. (nm)	RSD (%)	Average t_r_ (min)	Std. Dev. (min)	RSD (%)
Repeatability	30	33	4	13	12.9	0.1	1
	80	82	3	4	26.8	0.2	1
	170	169	5	3	48	1	3
	304	323	4	1	69	3	4
	490	527	4	1	95	3	3
Intermediate precision	30	30	5	18	13	0.1	1
	80	84	4	5	27	0.3	1
	170	167	4	3	49	2	4
	304	323	5	2	75	6	8
	490	531	5	1	96	3	3

Moreover, intermediate precision was studied for the same parameters as the repeatability. All of *F*-test and equal variance *t*-Student test showed that there were no significative differences between-days (*F*_exp_ < *F*_tab_) variances and between-days means (*t*_exp_ < *t*_tab_) from both *t*_r_ from UV detector and *D*_geo_ from MALS detector. On the other hand, both detectors precision had RSDs within the range of acceptance, 80–120%, and presented the same increasing/decreasing tendency explained in the repeatability (Table 2, bottom), indicating that there are no significant differences in between-days analysis.

#### Bias

To obtain the bias, in order to assess trueness, the NIST Std. mix was injected (*n* = 5), and the experimental *D*_geo_ was calculated using the separation method developed, obtaining a bias within the range of acceptance (80–120%) [35] for each one of the three standards: 99% for the 62 ± 3 nm Std. (calculated as 62 ± 2 nm), 100% for the 122 ± 3 nm Std. (calculated as 122 ± 2 nm), and 111% for the 345 ± 7 nm Std. (calculated as 381 ± 1 nm).

#### Linearity, LOD, and LOQ

The study of the linearity, limit of detection (LOD), and limit of quantification (LOQ) was performed independently for each detector [34]. As it can be seen in Table 3, a general increasing tendency in the LOD can be observed when the standard size decreases, which correlates with the low sensitivity of MALS toward lower size particles and the low of absorbance of small particles in the UV. For the LOQ, the results shown in Table 3 presented a tendency similar to the LOD where an increase of sensitivity and linear range can be observed as the particle size increases. The lowest standard size, 30 nm, was well detected and shown a linear tendency along all volume injections, but it could only be properly quantified with bias within range of acceptance between 1- and 2-µg injection (25–50 µL standard mix) due to the closeness with the void peak and its interference at the integration of the 30-nm standard peak. Furthermore, the UV detector showed the same LOQ than MALS detector (Table 3). However, when using the UV area (*V*_min_) instead of the MALS Intensity (V), the linearity range increased in most of the standards. The linear range for the standards presented the same tendency for the UV and MALS detectors, where the higher size standards showed a significant larger linear range than lower size standards.

**Table 3:** Limit of detection (LOD), limit of quantification (LOQ), and linearity range results for each NPL standard size (Std. mix) and for each detector (MALS and UV)

Std. D_geo_ (nm)	MALS			UV		
	LOD (µg)	LOQ (µg)	Linear range (µg)	LOD (µg)	LOQ (µg)	Linear range (µg)
30	0.31 ± 0.07	1.03 ± 0.01	1.0–2.0	0.8 ± 0.1	2.7 ± 0.2	3.00–8.00
80	0.05 ± 0.03	0.17 ± 0.03	0.20–2.00	0.11 ± 0.04	0.4 ± 0.1	0.40–10.00
170	0.003 ± 0.001	0.009 ± 0.001	0.04–0.40	0.04 ± 0.02	0.20 ± 0.09	0.20–2.00
304	0.04 ± 0.01	0.14 ± 0.08	0.10–0.80	0.04 ± 0.01	0.13 ± 0.05	0.10–2.00
490	0.04 ± 0.01	0.13 ± 0.09	0.10–0.60	0.06 ± 0.03	0.21 ± 0.09	0.20–1.50

Overall, both detectors showed a LOD below the range of study (< 0.2 µg, except for 30 nm) and the LOQ were also lower when compared to the overall range. Moreover, comparing both detectors, UV detectors area resulted in a much more efficient and reliable method for quantifying NPLs in aqueous matrixes giving larger linear ranges and lower LOD in most of the standards.

#### Resolution

**Fig. 3:**
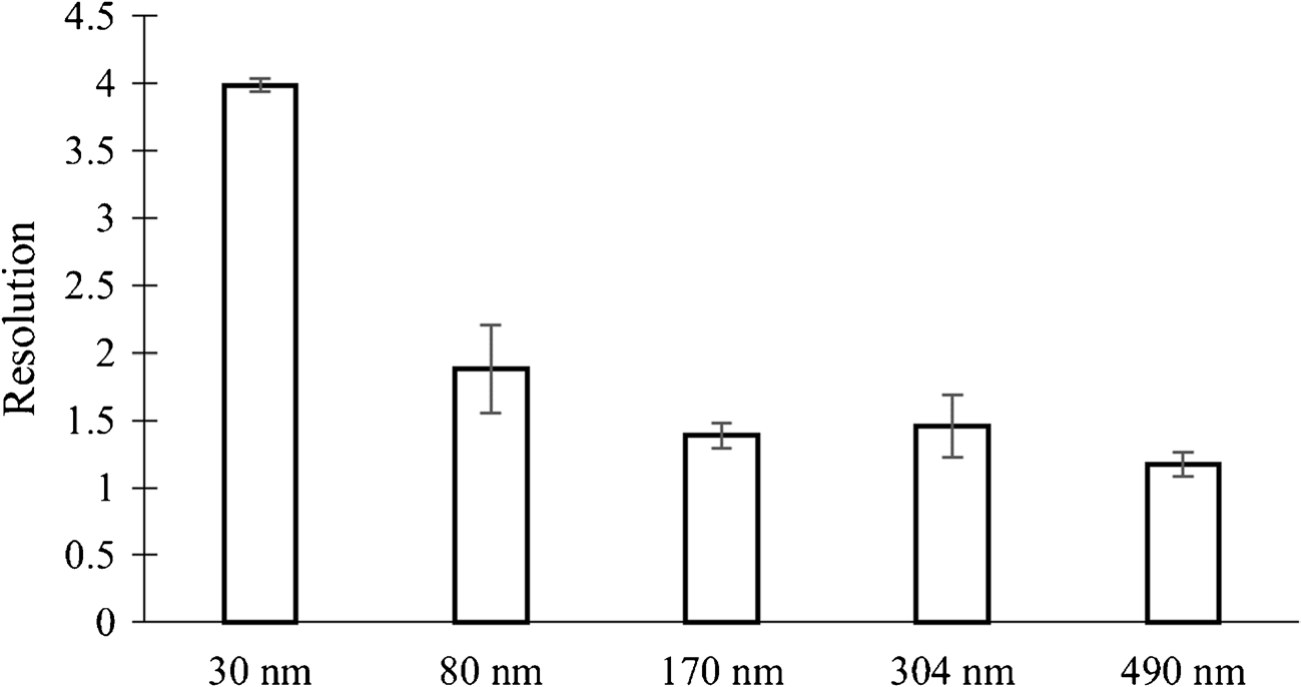
Resolution for each standard size (Std. mix)

The resolution of each peak resulted higher than one unit and close to 1.5. Baseline resolution is achieved when *R* = 1.5, but it is not generally expected to achieve baseline separation with the AF4 systems, so the target should be to achieve a resolution of at least *R* = 1.0 [37]. As can be seen in Fig. 3, the resolution decreases as the standard size increases, which is due to the method flow development. Since the flow decreases with an exponential function, at longer times, the flow decreases faster, which leaves less space between standards and, hence, less resolution. However, even having a lower resolution at longer times, it is still above the range of acceptance, as well as the bias and the precision results.

#### Membrane saturation study

Results of the *F* and *t*-Student tests, assuming equal variances for membrane saturation, indicated that the membrane saturation did not affect significantly to either the retention times studied in the UV detector nor the *D*_geo_ from the MALS detector, as *F*_exp_ < *F*_tab_ and *t*_exp_ < *t*_tab_. Moreover, precision and bias results concerning the Average Experimental *D*_geo_ obtained from the analysis of both membranes were within range of acceptance (Table 4), having the lowest precision at 30 nm. The same results were obtained when the retention times of the UV detector were studied (Table 4), showing no significant differences between the results obtained with a new membrane and a 100-h used membrane.

**Table 4:** Precision and bias results of the Std. *D*_geo_ determined with the MALS and UV detectors from the membrane saturation study. New membrane is expressed as NM, while the 100 h used membrane is expressed as UM

Std. D_geo_ (nm)	MALS detector						UV detector			
	Exp. D_geo_ NM (nm)	Exp. D_geo_ UM (nm)	Bias NM (%)	Bias UM (%)	Std. Dev (nm)	RSD (%)	Av. t_r_ NM (min)	Av. t_r_ UM (min)	Std. Dev (min)	RSD (%)
30	30	33	101	109	4	12	12.6	12.6	0.3	3
80	82	84	102	105	4	4	26.2	26.2	0.3	1
170	169	165	99	97	4	2	47	46	3	7
304	323	319	106	105	4	1	65.4	66.6	0.3	0.4
490	527	533	108	109	4	1	82	83	1	2

#### Injection volume study

Volume injection test study between 25 and 50 µL showed non-significant differences in the results concerning both, retention times of the UV detector and geometric radius obtained from the MALS detector, as *F*_exp_ < *F*_tab_ and *t*_exp_ < *t*_tab_. Furthermore, as shown in Table 5, diameters obtained with the two injections have accuracies within ranges of acceptance, observing no differences in the results obtained between the two injections. The same tendency is observed when studying the retention times of the UV detector, where the precision between retention time injections is less than 5% (Table 5). Overall, there are no significative differences between the two injection volumes, with the precision and bias of both detectors within ranges of acceptance, with the exception of the 30-nm standard due to its peak closeness to the void peak of the fractogram, since as higher volume is injected, longer is the tail of the void peak, increasing the integration effect due to the peak overlapping.

**Table 5:** Precision and bias results of the Std. *D*_geo_ determined with the MALS and UV detectors from the 25 and 50 µL volume injection study

Std. D_geo_ (nm)	MALS detector						UV detector			
	Exp. D_geo_ 25 µL (nm)	Exp. D_geo_ 50 µL (nm)	Bias 25 µL (%)	Bias 50 µL (%)	Std. Dev (nm)	RSD (%)	Av. t_r_ 25 µL (min)	Av. t_r_ 50 µL (min)	Std. Dev (min)	RSD (%)
30	29	32	97	105	2	6	12.4	12.2	0.2	2
80	82	83	102	103	1	1	26.2	26.2	0.3	1
170	167	169	98	99	1	1	44.4	44.1	0.3	1
304	320	324	105	107	3	1	65.8	66.1	0.6	1
490	500	519	102	106	13	3	83.5	83.5	0.3	0.3

## Conclusions

The results of this research show the successful development and validation of an AF4-MALS-UV methodology to determine NPL size in the range from 30 to 490 nm for standards in aqueous matrices. All the parameters studied agree and are within the acceptance ranges of the referenced publications and standards. Furthermore, this method provides accurate results within same day and between days showing an increasing sensitivity toward higher sizes in both detectors with a good linearity in all the range of interest. In addition, for the studied conditions, neither the injection volume nor the membrane saturation affected the veracity of the results, except for the 30-nm standard due to its closeness to the void peak and the interference generated from it when performing its integration. Hence, the use of AF4-MALS-UV optimized method results in an efficient and accurate way of determining the size of polydisperse NPL standards, being complementary to other composition characterization techniques such as pyrolysis–gas chromatrography-mass spectrometry or Fourier transformed infrared spectroscopy.

However, if the described method wants to be extrapolated to environmental samples, it must be further developed. Since the method has been optimized and validated for the analysis of polystyrene standards in aqueous solutions, other shapes than spheres that could be present in real environmental samples are not contemplated in the presented method. Moreover, due to the focus on the validation of a method for the size determination on standard mixtures in aqueous solutions, the possible matrix effect of real samples has not been evaluated.
